# Analysis of Endocannabinoid System in Rat Testis During the First Spermatogenetic Wave

**DOI:** 10.3389/fendo.2018.00269

**Published:** 2018-05-29

**Authors:** Marina Migliaccio, Giulia Ricci, Antonio Suglia, Francesco Manfrevola, Ken Mackie, Silvia Fasano, Riccardo Pierantoni, Teresa Chioccarelli, Gilda Cobellis

**Affiliations:** ^1^Department of Experimental Medicine, Sez. Bottazzi, Università degli Studi della Campania “L. Vanvitelli”, Naples, Italy; ^2^Department of Experimental Medicine, Laboratorio di Istologia ed Embriologia, Università degli Studi della Campania “L. Vanvitelli”, Naples, Italy; ^3^Department of Psychological and Brain Sciences, Indiana University, Bloomington, IN, United States

**Keywords:** testis, endocannabinoid system, spermatogenesis, germ cells, CB1, CB2

## Abstract

Endocannabinoids are lipid mediators, enzymatically synthesized and hydrolyzed, that bind cannabinoid receptors. Together with their receptors and metabolic enzymes, they form the “endocannabinoid system” (ECS). Anandamide (AEA) and 2-arachidonoylglycerol (2-AG) are the main endocannabinoids studied in testis. In this study, using the first wave of spermatogenesis as an *in vivo* model to verify the progressive appearance of germ cells in seminiferous tubules [i.e., spermatogonia, spermatocytes, and spermatids], we analyzed the expression of the main enzymes and receptors of ECS in rat testis. In particular, the expression profile of the main enzymes metabolizing AEA and 2-AG as well as the expression of cannabinoid receptors, such as CB1 and CB2, and specific markers of mitotic, meiotic, and post-meiotic germ cell appearance or activities have been analyzed by RT-PCR and appropriately correlated. Our aim was to envisage a relationship between expression of ECS components and temporal profile of germ cell appearance or activity as well as among ECS components. Results show that expression of ECS components is related to germ cell progression. In particular, CB2 and 2-AG appear to be related to mitotic/meiotic stages, while CB1 and AEA appear to be related to spermatogonia stem cells activity and spermatids appearance, respectively. Our data also suggest that a functional interaction among ECS components occurs in the testis. Indeed, *in vitro*-incubated testis show that AEA-CB2 activity affects negatively monoacylglycerol-lipase levels *via* upregulation of CB1 suggesting a CB1/CB2-mediated relationship between AEA and 2-AG. Finally, we provide the first evidence that CB1 is present in fetal gonocytes, during mitotic arrest.

## Introduction

Spermatogenesis is a complex biological process including proliferation, meiosis, and differentiation of germ cells, from spermatogonia (SPG) stem cells to spermatozoa (SPZ). The proliferation phase starts during the embryonic development (1). In rat, proliferating primordial germ cells reach the genital ridges at 10.5 *days post coitum* (*dpc*). They colonize the differentiating gonad and, triggered by the testicular microenvironment (2–4), start proliferating to give rise to gonocytes. Proliferation ends at 18.5 dpc, when gonocytes enter G1/G0 mitotic arrest (5). At 3 *days post partum* (*dpp*), gonocytes resume the cell cycle and, after DNA synthesis, are blocked in G2 phase. On 4–5 *dpp*, germ cells migrate toward the basement of seminiferous tubules and enter mitosis establishing the initial pool of unipotent SPG stem cells (5). This self-renewal activity produces undifferentiated SPG (i.e., proSPG) which differentiate (commitment in type A1 SPG) and proliferate (A1-A4-B SPG) before starting meiosis as spermatocytes (SPC) (around 10–12 *dpp*). Spermatids (SPT) are produced (around 26 *dpp*), then they differentiate in SPZ (around 45 *dpp*) through a dramatic reorganization involving both the nuclear and cytoplasmic compartments (6, 7).

Spermatogenesis is finely regulated by various hormones and factors that act through endocrine, autocrine, and paracrine pathways (8, 9–20). Among these, endogenous cannabinoids (i.e., endocannabinoids) are lipid mediators emerging as modulators of spermatogenesis (21–25) and reproduction (26–28). The endocannabinoids are produced “on demand” from membrane phospholipids. They bind and activate type-1 (CB1) and type-2 (CB2) cannabinoid receptors (29, 30) mimicking some effects of Δ*9*-tetrahydrocannabinol, the psychoactive component of the *Cannabis sativa*. Arachidonoylethanolamine (i.e., anandamide, AEA), and 2-arachidonoylglycerol (2-AG) are the main endocannabinoids identified in vertebrates (31, 32); their synthesis is mainly catalyzed by NAPE-hydrolyzing phospholipase D (NAPE-PLD) (33) and *sn*-1-DAG-lipase (DAGL) (34), respectively. Their degradation is controlled by two specific enzymes: the fatty acid amide hydrolase (FAAH) that preferentially degrades AEA (2-AG at less extent) while the monoacylglycerol-lipase (MAGL) hydrolyzes 2-AG (35, 36). The biosynthetic/hydrolyzing enzyme balance regulates the endocannabinoid content determining the appropriate “tone,” which is critical for many physiological processes (37, 38), including reproduction (39, 40). Receptors, endocannabinoid membrane transporter, ligands, and their metabolic enzymes form the “endocannabinoid system” (ECS) (41).

Many studies have been carried out to analyze expression and function of ECS in the male reproductive tract (42–51). The components of this system have been identified in mammalian and non-mammalian testes, in somatic and germ cells, from SPG to SPZ (21, 43, 44, 46, 49–56). CB1 has been localized in differentiating/mature adult Leydig cells, in SPT and SPZ and it seems to be positively related to (i) steroidogenesis (20–22, 56) and differentiation (49) of Leydig cell, (ii) chromatin remodeling (7, 57) of SPT, and (iii) maturation and quality of SPZ (44, 46, 47, 51, 52, 54, 55, 58). Interestingly, in rat testis, a weak and stage-specific expression of CB1 has been observed in Sertoli cells (22, 49) when SPT appear in seminiferous tubules. Study about CB1 activity in these cells is lacking. CB2 is highly expressed in murine SPG and SPC (a weak signal appears in SPT), and also in Sertoli cells isolated from 7, 16, and 18 *dpp* old mouse (43, 50). Data obtained from mice show that CB2, autocrinally activated by 2-AG, has a role in SPG proliferation and meiotic entry (50, 59). Indeed, 2-AG levels are high in SPG and dramatically decrease in SPC and round SPT (50). On the contrary, AEA levels mainly remain unchanged in germ cells, from SPG-to-SPT. However, Leydig and Sertoli cells contain AEA (21, 43). In Sertoli cells, a complete biochemical machinery to transport/degrade and to bind AEA *via* CB2 is present (43). AEA shows pro-apoptotic effects on Sertoli cells. Lower levels of AEA correlate with Sertoli cell age and higher levels of FAAH suggesting a protective and pro-survival role of FAAH in these cells (48).

In this study, using the first wave of spermatogenesis as an *in vivo* model to study the progressive appearance of germ cells in seminiferous tubules (i.e., SPG, SPC, and SPT), we analyzed the expression of the main enzymes and receptors of ECS in rat testis. Our aim was to study the expression ECS components in testis during progressive appearance germ cells and to find a relationship between expression of ECS components and germ cell activity (i.e., mitosis, meiosis, SPT–SPZ differentiation).

## Materials and Methods

### Experimental Animals

Experiments were approved by the Italian Ministry of Education and the Italian Ministry of Health. All methods and all animal procedures were performed in accordance with the relevant guidelines and regulations by National Research Council’s for Care and Use of Laboratory Animals (NIH Guide).

Sprague-Dawley rats (*Rattus norvegicus*, from Charles River Laboratories, Lecco, Italy) and mice (*Mus musculus*) genetically deleted of CB1 in heterozygous conditions (CB1^+/−^), provided by Prof. Ledent [knock out has been obtained by replacing the first 233 codons of CB1 gene with PGK-Neo cassette (60)], were kept in a room with controlled temperature (22 ± 2°C), ventilation, and lighting (12-h light/dark cycles) and were maintained on a standard pellet diet with free access to water. Male and female heterozygous CB1 mice will be bred on a CD1 background (Charles River Laboratories, Lecco, Italy) to expand colony, then used to generate WT and CB1^+/−^ male mice available for *in vitro* experiments.

Pregnant rats were sacrificed at 19.5 *dpc* and fetal testes from male embryos (*n* = 4) were collected and fixed for immunohistochemistry. Male rats were killed at 1, 7, 14, 21, 27, 31, 35, 41, 60 and 90 *dpp* and testes rapidly removed, frozen on dry ice, and properly stored for RT-PCR analyses. At least three animals/age were analyzed and at least one testis/animal separately processed for RT-PCR analysis.

Adult (6–8 months) male mice CB1^+/−^ were killed by CO_2_ asphyxia, and testes processed for *in vitro* tissue incubation. Each experimental analysis included at least three testis/experimental group from different animals, and each testis/animal was separately analyzed.

### Total RNA Preparation

Total RNA was isolated from rat testes using TRIZOL^®^ Reagent (Invitrogen Life Technologies, Paisley, UK) according to the manufacturer’s recommendations. To remove potential contamination of genomic DNA, RNA aliquots (10 µg) were treated with 2 U DNase (RNase-free DNase I, Ambion, Thermo Fisher Scientific, MA, USA) according to the manufacturer’s recommendations. Purity and integrity of RNA samples were determined as previously reported (61).

### RT-PCR Analysis

Semi-quantitative RT-PCR is a specific technique although less sensitive as compared with Real Time PCR. This allows one to appreciate gene expression differences, just when these are evident and stable. Using RNA from whole testis, we consider this method to robustly run correlation tests of gene expression. To reinforce our methodological choice, we currently compared our data with those reported by Grimaldi characterizing ECS components in isolated mouse germ cells (50).

As already reported (49), total RNA (2 µg) was used to synthesize cDNA in 20 µl mix containing: 0.5 µg oligo dT, 10 mM dNTP, 0.01 M DTT, 1× first strand buffer (Invitrogen Life Technologies, Paisley, UK), 40 U RNase Out (Invitrogen Life Technologies, Paisley, UK), 200 U SuperScript-III RNaseH-Reverse Transcriptase (Invitrogen Life Technologies, Paisley, UK). As a negative control, total RNA (2 µg) was incubated in the previous mix without adding Reverse Transcriptase enzyme (RT− cDNA sample). PCR was carried out using 2 µl cDNA and 10 pmol of the appropriate primers in a PCR mix [0.2 mM dNTP, 1× PCR buffer (Invitrogen Life Technologies), 1.5 mM MgCl_2_, 1.25 U Taq Polymerase (Invitrogen Life Technologies, Paisley, UK)], using an Applied Biosystem Thermocycler. Possible residual genomic DNA was evaluated using *Actin* primers by RT− cDNA amplification (at 35 circles) while possible contamination among samples was excluded using samples prepared with 1 µl water (H_2_O samples, negative control) in place of cDNA. Except for CB1 gene, primers were specifically designed on different exons (i.e., amplicons would span an intron if genomic DNA was amplified). Their sequences and PCR program are reported in Table 1. Each cDNA was amplified in duplicate or triplicate, in three independent experiments, and analyzed by electrophoresis on an agarose gel. Signals were quantified by densitometry analysis and graphed as optical density (OD) values normalized relatively to *Actin* (normalized OD values) amplified at 25 circles by excluding the saturation phase.

**Table 1:** Primer sequences (S: sense; AS: antisense), annealing temperature (Tm) and cycles number used to analyse testicular levels of *Nape-pld, Faah, Magl, Dagl, Cb1, Cb2, Cxcl12, Mlh3, Hsp70t*, and *Actin* mRNA by RT-PCR.

Gene primers	Sequences 5′–3′	Tm (°C)	No. cycles	Product size(bp)
*Neomycin* S *Neomycin* AS	gatccagaacatcaggtagg aaggaagggtgagaacagag	56	35	521
*Cb1 S *Cb1 AS	catcatcacagatttctatgtac gaggtgccaggagggaacc	56	35	366
*Nape-pld* S *Nape-pld* AS	agatggctgataatggagaa ttctcctcccaccagtc	56	35	463
*Faah* S *Faah* AS	ggaagtgaacaaagggacca actgacattggcggcagcat	60	35	220
*Dagl* S *Dagl* AS	cacgaggcctgctccctgaa gtgagccagacgatgcccac	60	35	218
*Magl* S *Magl* AS	ggtcaatgcagacggacagt atggagtggcccaggaggaa	64	35	303
*Cb1* S *Cb1* AS	atcctagatggccttgcaga taaagttctccccacactgga	56	35	300
*Cb2* S *Cb2* AS	ggcagcgtgaccatgacctt gacgtgaggttggccaagac	60	35	406
*Cxcl12* S *Cxcl12* AS	ccagtcagcctgagctac gtctactggaaagtcctttg	58	35	235
*Mlh3* S *Mlh3* AS	gactaagagtattgtggagg gcacactgaaatggcagct	58	35	213
*Hsp70t* S *Hsp70t* AS	ggtgatgagggtctgaag gggtgggggtgtgaaaac	52	35	421
*Actin* S *Actin* AS	ctcttccagccttccttcct ctgcttgctgatccacatc	60	25	298

In table are also indicate primer sequences for neomycin-cassette and CB1 gene (*) used for genotyping CB1^+/−^ mice. Product size indicate base pairs of the amplified fragments.

The genotype of CB1^+/−^ testes was verified by PCR analysis of genomic DNA using specific primers for neomycin-cassette and CB1 gene (Table 1).

### Immunocytochemistry (ICC)

Testes from 14.5 and 19.5 *dpc* rat embryos and 90 *dpp* rat were fixed overnight in Bouin’s solution and embedded in paraffin using standard procedures, as previously described (38, 62). Microtome sections (5-µm thick) were stained with hematoxylin & eosin or incubated overnight with primary antibody solution [rabbit anti-human N-terminus CB1; diluted 1:500 (63)], rabbit anti-human NAPE-PLD (diluted 1:100 #10305, Cayman, MI, USA), rabbit anti-rat FAAH (diluted 1:500; #101600, Cayman, MI, USA), rabbit anti-rat MAGL (diluted 1:100, #24701, Abcam, Cambridge, UK) in 0.01 M PBS, 1% Triton-X100, 10% BSA (49). Immunoreactivity was revealed using the avidin–biotin complex system and H_2_O_2_/DAB (3,3′-diaminobenzidine-tetrahydrochloride) as substrate/chromogen. The specificity of immunoreaction has already been demonstrated (27, 28, 49, 51) and here routinely checked by omitting primary antibody. Sections were observed under a light microscope (Leica CTR500) and images captured using a high resolution digital camera (Leica DC300F).

### Correlation Curves

Data of gene expression (normalized OD values, from three to five independent RT-PCR analyses) concerning ECS components (*Nape-pld, Faah, Dagl, Magl, Cb1, Cb2 mRNA*, as well as *Dagl/Magl* ratio *mRNA*, here used as index of intra-testicular 2-AG levels) and germ cell activity markers [chemokine (C-X-C motif) ligand 12 (*Cxcl12*); mutL homolog 3 (*Mlh3*); and a mouse testis-specific heat shock 70 protein (*Hsp70t*) *mRNA*] were conveniently compared, within specific time frames of the first spermatogenic wave, using the Excel built-in distribution functions available in Microsoft Office. The “*r*” value was considered to establish the test significance. The range −1 ≤ *r* ≤ 1 established negative or positive correlation.

### *In Vitro* Testes Incubations and Western Blot Analysis

Anandamide and AM630 (6[6-iodo-2-methyl-1-[2-(4-morpholinyl)ethyl]-1H-indol-3-yl](4-methoxyphenyl)-methanone), a selective CB2 inverse agonist, were obtained from Sigma-Aldrich (Milan, Italy). Both drugs were of the purest analytical grade and each were dissolved in ethanol (i.e., AEA) or dimethylsulfoxide (DMSO; i.e., AM630) according to the manufacturer’s instructions.

CB1^+/−^ testes with a feeble notch in tunica albuginea (*n* = 3 or 4 for experimental group) were incubated in PBS (6 ml) for 90 min with vehicle (0.015% ethanol plus 0.05% DMSO according to relative compound concentrations; control group) or with AEA (1 µM) ± AM630 (10 µM). Ethanol (0.0015%) and DMSO (0.05%) were added in each experimental group. AM630 was always added 30 min before AEA at a concentration useful to affect CB2 activity as previously reported in cell cultures and *in vitro* testis (57, 64, 65) as well as in *in vivo* mice (59). Each testis/experimental group was separately homogenized in RIPA buffer [PBS, pH 7.4, 10 mM dithiothreitol, 0.02% sodium azide, 0.1% SDS, 1% Nonidet P-40, 0.5% sodium deoxycholate, in the presence of protease inhibitors (10 µg/ml of leupeptin, aprotinin, pepstatin A, chymostatin, and 5 µg/ml of TPCK)], as already reported (54) and analyzed by Western blot. Briefly: proteins (90 µg) were separated by SDS-PAGE (10% acrylamide) and transferred to polyvinylidene difluoride membrane (GE Healthcare) at 280 mA for 2.5 h at 4 C. Membrane was cut at 50-kDa level. The upper and lower filters were treated for 3 h with blocking solution [5% nonfat milk, 0.25% Tween-20 in Tris-buffered saline (TBS, pH 7.6)] and then separately incubated overnight, at 4°C in TBS-milk buffer (TBS pH 7.6, 3% nonfat milk) with different primary antibody [MAGL, diluted 1:500, code ab24701 from Abcam, Cambridge, UK; ERK1/2, diluted 1:1,000, code sc-154 from Santa Cruz Biotechnology, Inc., Heidelberg, Germany; CB1 C-terminal (46), diluted 1:1,000]. After washing in 0.25% Tween20-TBS, filters were incubated with 1:1,000 horseradish peroxidase-conjugated rabbit IgG (Dako Corp., Milan, Italy) in TBS-milk buffer and then washed again. The immune complexes were detected using the enhanced chemiluminescence-Western blotting detection system (Amersham ECL Western Blotting Detection Reagent, cod: RPN2106, GE Healthcare). Signals were quantified by densitometry analysis, adjusted relatively to ERK1/2 levels and graphed as OD fold change (mean ± SEM).

The specificity of immunoreaction has already been demonstrated (27, 28, 49, 51) and here routinely checked by omitting primary antibody (data not shown).

### Statistical Analysis

Student’s *t*-test or ANOVA followed by Duncan’s test for multi-group comparison were performed, where appropriate, to evaluate the significance of differences. Data were expressed as the mean ± SEM.

## Results

### Analysis of ECS Components During the First Wave of Spermatogenesis

The presence of ECS components has been investigated in rat testis by RT-PCR analyses. In particular, the expression profile for *Faah, Nape-pld, Magl, Dagl*, and *Cb2 mRNA* has been analyzed from 7 to 60 *dpp*. The expression of *Cb1* in rat testis during the first spermatogenic wave has already been investigated by our group (49) and here just analyzed during perinatal period.

**Figure 1:**
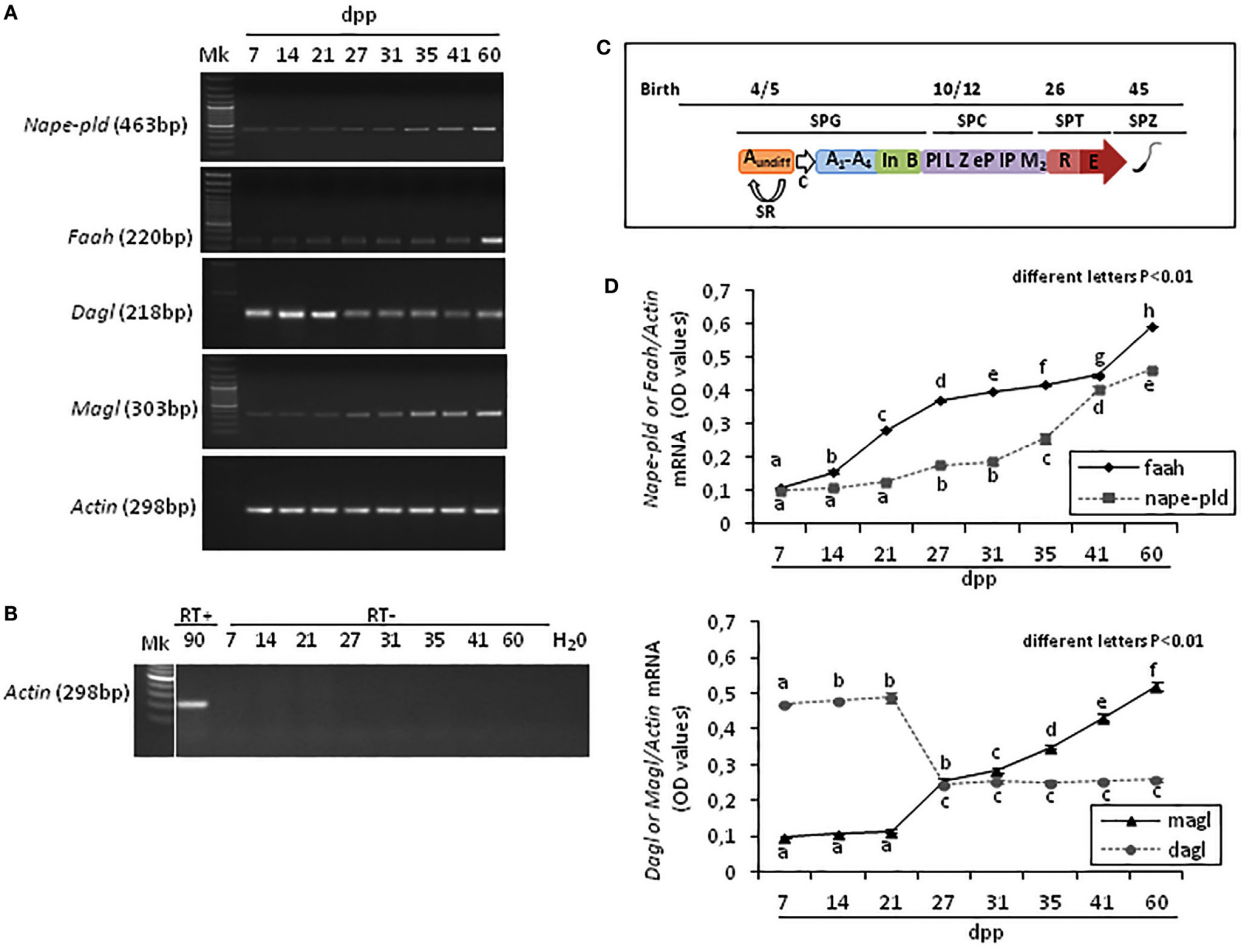
RT-PCR analysis of the main AEA and 2-arachidonoylglycerol metabolizing enzymes in rat testis during the first wave of spermatogenesis. Representative images of RT-PCR analyses showing *Nape-pld, Faah, Dagl, Magl*, and *Actin* mRNA expression in rat testes from 7 to 60 *days post partum* (*dpp*) **(A)**. Representative agarose gel image of RT + cDNA (testes from 90 *dpp* rats), RT− cDNA (testes from 7 to 60 *dpp* rats) and water (H_2_O) analyzed by PCR using actin primers **(B)**. Gene expression was quantified by densitometry analysis and graphed relatively to germ cells appearance or activities **(C)**. Values for *Nape-pld, Faah, Dagl*, and *Magl* signals were normalized against *Actin* and are expressed as OD values **(D)**. All data are expressed as the mean ± SEM. Different letters indicate statistically significant differences (*p* < 0.01). Abbreviations: SPG, spermatogonia; SPC, spermatocytes; SPT, spermatids; SPZ, spermatozoa; A_undiff_, undifferentiated-SPG; A_1_-A_4_, differentiating type-A_1_-A_4_ SPG; In, differentiating intermediate-SPG; B, differentiating type-B SPG; Pl, preleptotene SPC; L, leptotene SPC; Z, zygotene SPC; eP, early pachytene SPC; lP, late pachytene SPC; M_2_, secondary SPC; R, round SPT; E, elongated SPT; SR, self-renewal; C, commitment.

Results show that *Faah, Nape-pld, Magl, Dagl*, and *Actin* were constantly present in rat testis from 7 to 60 *dpp*. The Figure 1A shows one band of the predicted size for each transcript (see specific amplicons in Table 1). The Figure 1B shows representative agarose gel image of RT+ (testes from 90 *dpp* rats) and RT− (testes from 7 to 60 *dpp* rats) cDNA analyzed by PCR using actin primers.

Densitometry analysis of signals, normalized relatively to Actin, and graphed relatively to temporal profile of germ cells appearance or activities (Figure 1C), revealed specific fluctuations of gene expression (Figure 1D). At 7 *dpp, Faah* and *Nape-pld* were poorly expressed. Thereafter, *Faah mRNA* progressively and significantly increased up to 60 *dpp* (*p* < 0.01). *Nape-pld mRNA* was low with no significant increase from 7 to 21 *dpp*. Later, a significant increase was observed at 27 *dpp* (*p* < 0.01) with a progressive higher expression from 31 *dpp* forward. Interestingly, *Faah mRNA* was constantly higher when compared with *Nape-pld* suggesting that *Faah* expression was mainly activated during the spermatogenic wave to downregulated intra-testicular AEA levels, except to 7 and 41 *dpp* when matching *Nape-pld/Faah mRNA* values were observed suggesting intra-testicular increase of AEA. *Dagl mRNA* levels were high at 7–21 *dpp* and drastically reduced from 27 to 60 *dpp* (*p* < 0.01). *Vice versa, Magl mRNA* levels were low at 7–21 *dpp* and a progressive higher expression was observed from 27 *dpp* forward (*p* < 0.01), suggesting few intra-testicular 2-AG.

The presence of CB1 in fetal testis was studied by ICC at 14.5 and 19.5 *dpc* during proliferation and mitotic G1 arrest of germ cells, while the fluctuations of *Cb1* and *Cb2 mRNA* were analyzed, using RT-PCR, during early *post-natal* period (from 1 to 14 *dpp*) and during all the spermatogenic wave (from 7 to 60 *dpp*), respectively.

**Figure 2:**
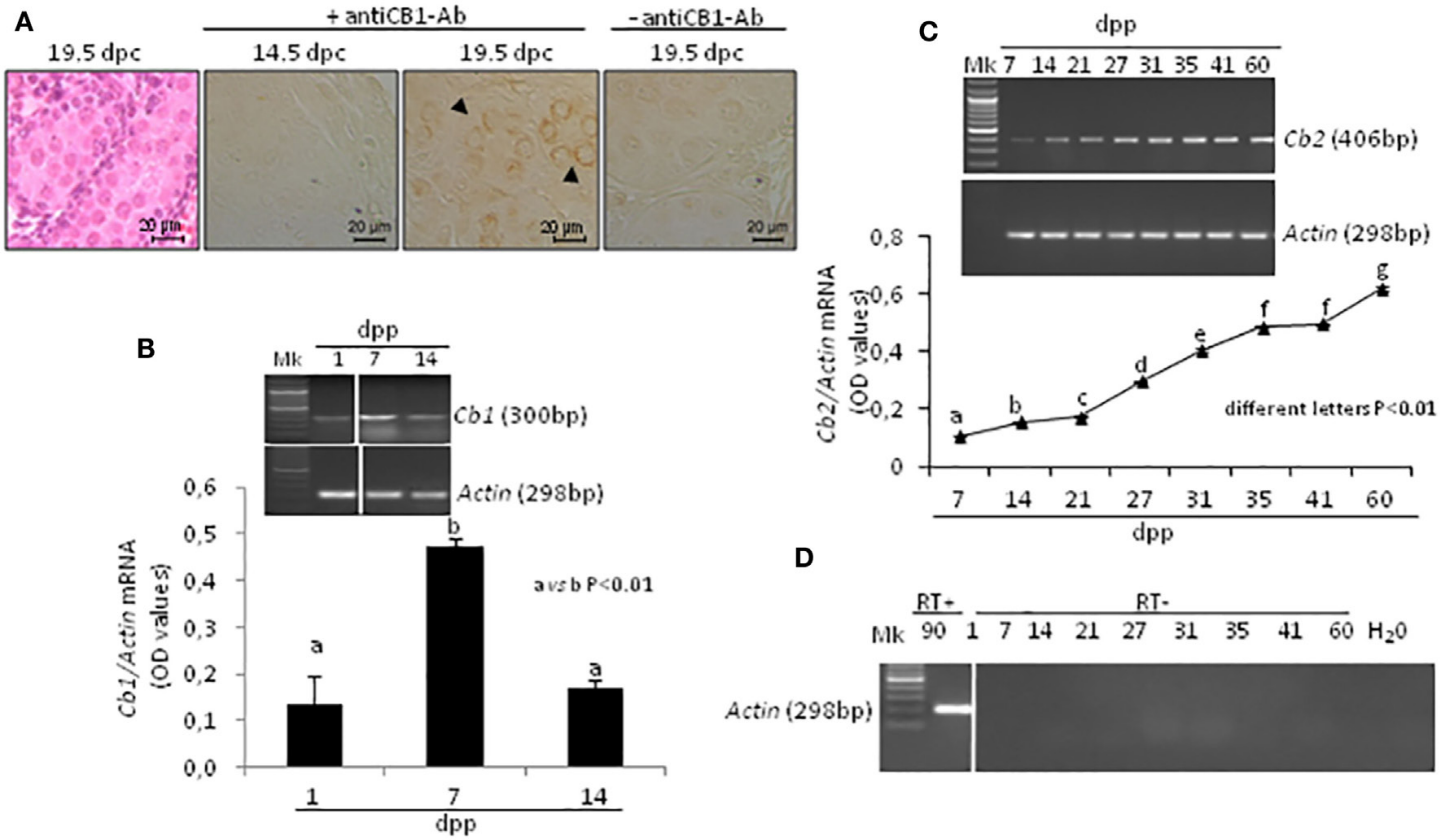
Expression of cannabinoid receptors in fetal and post-natal rat testis. Representative fetal testis section from 19.5 *days post coitum* (dpc) old rats stained with hematoxylin & eosin and immunostained for CB1. Arrowheads indicate immunopositive gonocytes. Scale bar: 20 µm **(A)**. Representative images of RT-PCR analyses showing *Cb1* and *Actin* mRNA expression in rat testes from 1-to-14 *days post partum* (*dpp*). *Cb1* expression was quantified by densitometry analysis, normalized against *Actin* signals and expressed in OD values **(B)**. Representative images of RT-PCR analyses showing *Cb2* and *Actin* mRNA expression in rat testes, from 7 to 60 *dpp*. *Cb2* expression was quantified by densitometry analysis, normalized against Actin signals and expressed in OD values **(C)**. Representative agarose gel image of RT + cDNA (testes from 90 *dpp* rats), RT− cDNA (testes from 1 to 60 *dpp* rats) and water (H_2_O) analyzed by PCR using actin primers **(D)**. All data are reported as the mean ± SEM. Different letters indicate statistically significant differences (*p* < 0.01). The white bar in the agarose gel image indicates cropped figures from same gel.

In fetal testis, CB1 was absent at 14.5 *dpc* and present at 19.5 *dpc*. The protein was specifically related to gonocytes (Figure 2A). Specificity of immunoreactions has already been demonstrated and here checked again by omitting the primary antibody.

Transcripts for *Cb1, Cb2*, and *Actin* mRNA were present in testis. A single band of the predicted size for each transcript (see specific amplicons in Table 1) was obtained (upper panels Figures 2B,C). Densitometry analysis of signals, normalized relatively to *Actin*, revealed fluctuations of gene expression specifically related to each receptor (lower panels Figures 2B,C). *Cb1 mRNA* was more highly expressed at 7 *dpp* (a vs b, *p* < 0.01) as compared to 1 and 14 *dpp* (Figure 2B). *Cb2 mRNA* was feebly present at 7 *dpp*. Except to 41 *dpp*, its expression significantly and steadily increased from 7 to 60 *dpp* (different letters *p* < 0.01), with higher increases from 27 *dpp* onward (Figure 2C). The Figure 2D shows representative agarose gel image of RT+ (testes from 90 *dpp* rats) and RT− (testes from 1 to 60 *dpp* rats) cDNA analyzed by PCR using *Actin* primers.

### Germ Cell Progression During the First Wave of Spermatogenesis

**Figure 3:**
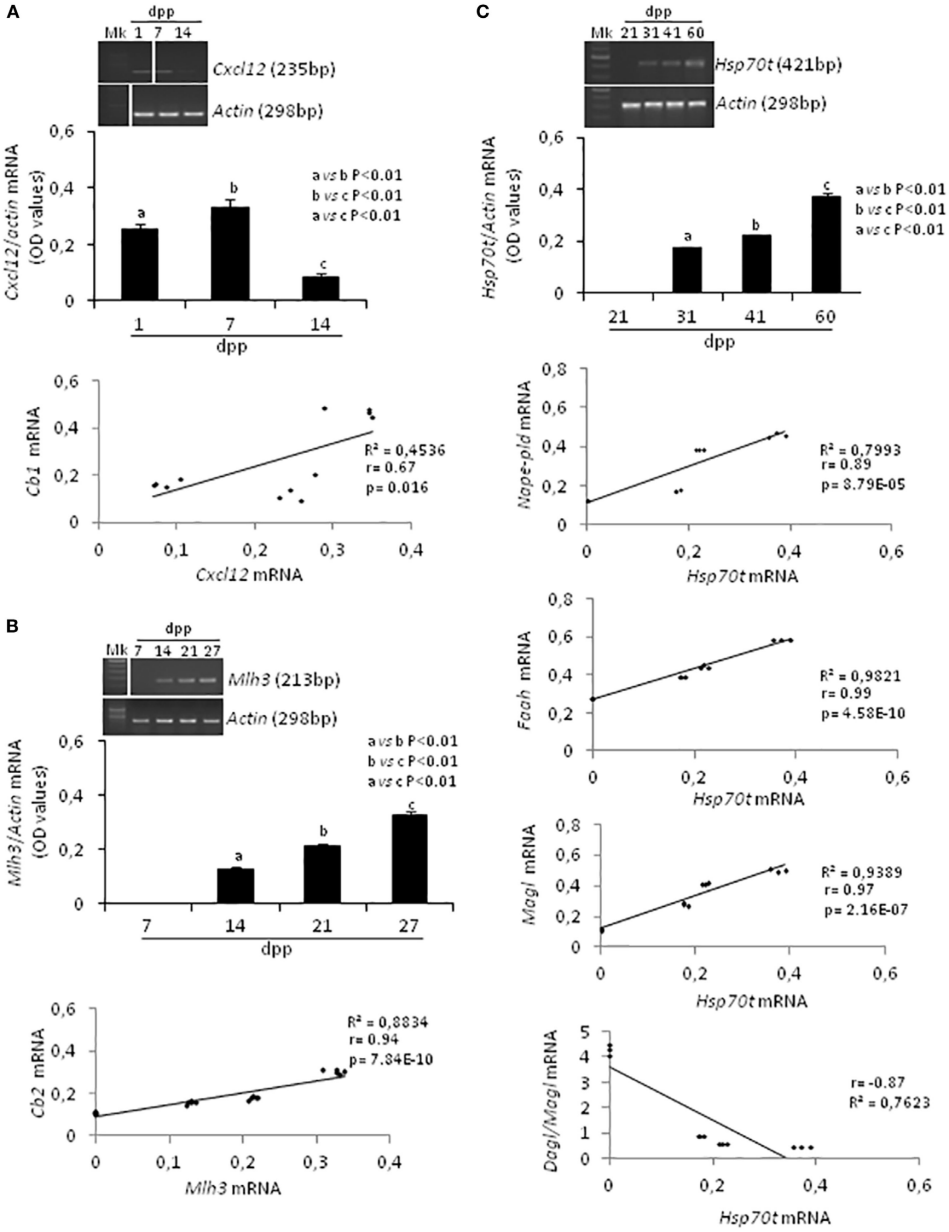
RT-PCR analyses of specific markers related to the activity and/or presence of germ cells at specific times during the first spermatogenetic wave and relative correlation analyses. Representative images of RT-PCR analyses showing *Cxcl12* and *Actin* mRNA expression in rat testis from 1 to 14 *days post partum* (*dpp*). *Cxcl12* expression was quantified by densitometry analysis and normalized against *Actin* signals. Values, expressed as OD have been used for correlation analysis between *Cxcl12* and *Cb1* mRNA expression **(A)**. Representative images of RT-PCR analyses showing *Mlh3* and *Actin* mRNA expression in rat testis from 7 to 27 *dpp*. *Mlh3* expression was quantified by densitometry analysis and normalized against Actin signals. Values, expressed in OD, have been used for correlation analysis between *Mlh3* and *Cb2* mRNA expression **(B)**. Representative images of RT-PCR analyses showing *Hsp70t* and *Actin* mRNA expression in rat testis from 21 to 60 *dpp*. *Hsp70t* expression was quantified by densitometry analysis and normalized against *Actin* signals. Values, expressed as OD, have been used for correlation analysis between *Hsp70t* and *Nape-pld* or *Faah* or *Magl* or *Dagl*/*Magl* mRNA expression **(C)**. Different letters indicate statistically significant differences (*p* < 0.01). The white bars in the agarose gel images indicate cropped figures from same gel.

Specific molecular markers related to proliferation (66), meiosis (67), and differentiation (68) of germ cells were evaluated by RT-PCR analysis and conveniently correlated to ECS component expression during specific time frame of the first spermatogenetic wave. In particular, *Cxcl12* was used as marker of SPG stem cell pool maintenance activity (2–14 *dpp*); *Mlh3* and a mouse testis-specific *Hsp70t*, were used as markers of appearance and presence of SPC (7–27 *dpp*) and SPT (21–60 *dpp*), respectively. *Actin* was analyzed as housekeeping gene. Figure 3 shows (i) representative images of RT-PCR analyses, (ii) graphs relative to densitometry analyses of signals (Figures 3A–C, upper panels), and (iii) appropriate correlation curves (Figures 3A–C, lower panels).

RT-PCR analysis confirmed the presence of *Cxcl12, Mlh3, Hsp70t*, and *Actin* mRNA as expected during the time frames specifically examined (i.e., mitotic, meiotic, differentiation phases). A single band of the predicted size for each transcript (see specific amplicons in Table 1) was obtained (upper panels Figures 3A–C). Densitometry analysis of signals, normalized relatively to *Actin*, revealed fluctuations of expression specifically related to each gene.

*Cxcl12 mRNA* was significantly higher at 7 *dpp* (*p* < 0.01) when compared with 1 and 14 *dpp* (Figure 3A, upper panels). The expression profiles of *Cxcl-12* and *Cb1* from 1 to 14 *dpp* were positively and significantly correlated (*r* = 0.67, *p* < 0.05; Figure 3A lower panel).

At 7 *dpp, Mlh3 mRNA* was absent. This first appeared at 14 *dpp* and increased steadily over the time (Figure 3B, upper panels). The expression profiles of *Mlh3* and *Cb2*, from 7 to 27 *dpp*, were positively and significantly correlated (*r* = 0.94, *p* < 0.01; Figure 3B, lower panel).

*Hsp70t mRNA* was absent at 21 *dpp*. It first appeared at 31 *dpp* and its expression levels increased progressively over time (Figure 3C, upper panels). The correlation analyses show that the expression profiles of *Hsp70t*, from 21 to 60 *dpp*, correlated positively and significantly with those of *Nape, Faah*, and *Magl* (0.9 < *r* < 1, *p* < 0.01) and negatively with the *Dagl/Magl mRNA* ratio (*r* = −0.87) (Figure 3C, lower panels).

### Correlation Analyses Among ECS Components

**Figure 4:**
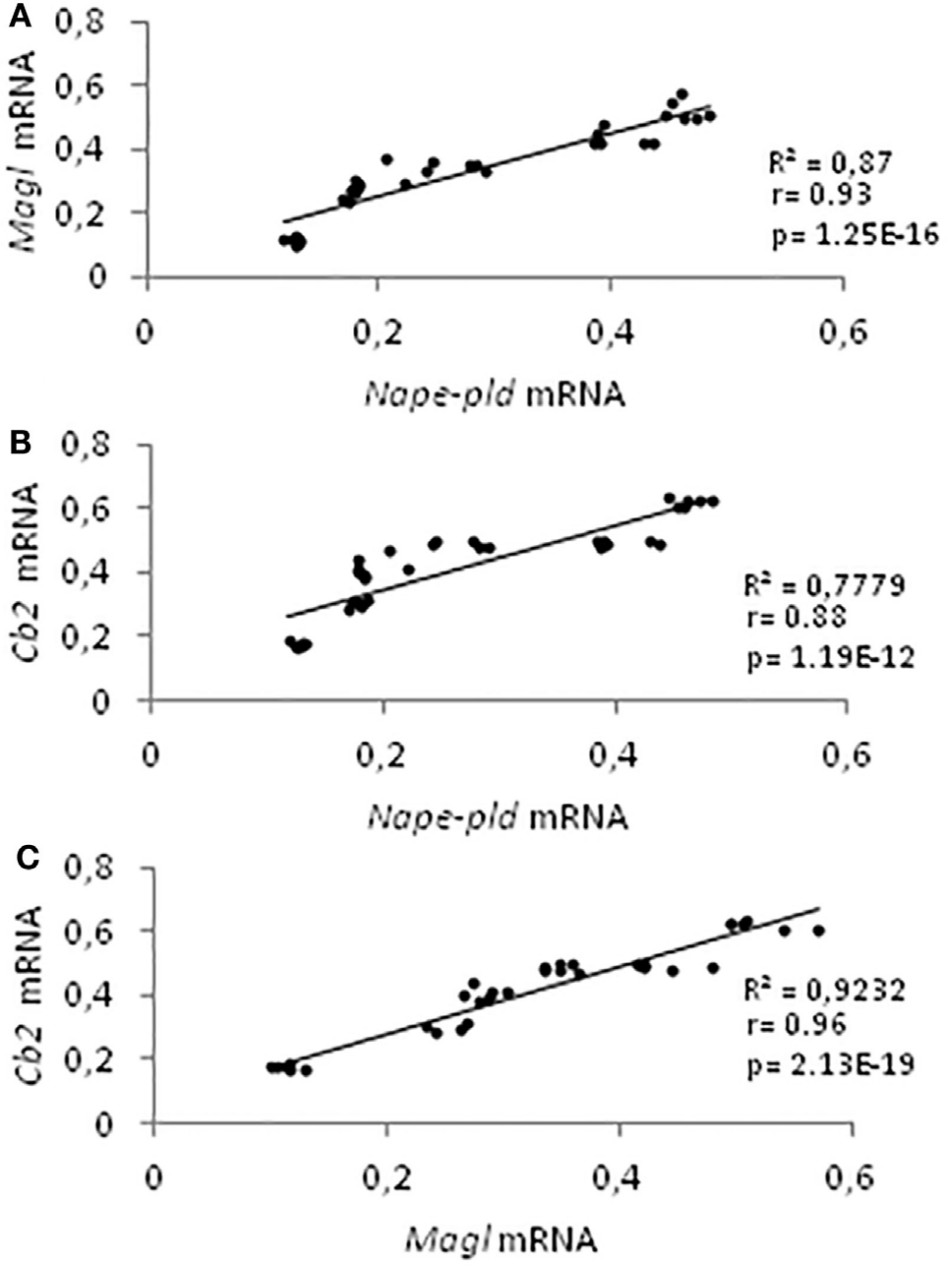
Correlation analyses among endocannabinoid system components. Correlation analyses between Nape-pld and Magl **(A)**, Nape-pld and Cb2 **(B)**, Magl and Cb2 **(C)** mRNA expression, from 21 to 60 *days post partum*.

Data on gene expression for *Nape-pld, Faah, Dagl, Magl*, and *Cb2*, from 21 to 60 *dpp*, have been used for correlation studies. Results show that the expression profiles of *Nape-pld, Magl*, and *Cb2 mRNA* were positively and significantly correlated each other’s (0.88 < *r* < 0.96; *p* < 0.01; Figures 4A–C). No significant correlation was found when the analysis was carried out using *Faah* or *Dagl mRNA* profiles (data not show).

### Localization of ECS Components in Germ Cells

**Figure 5:**
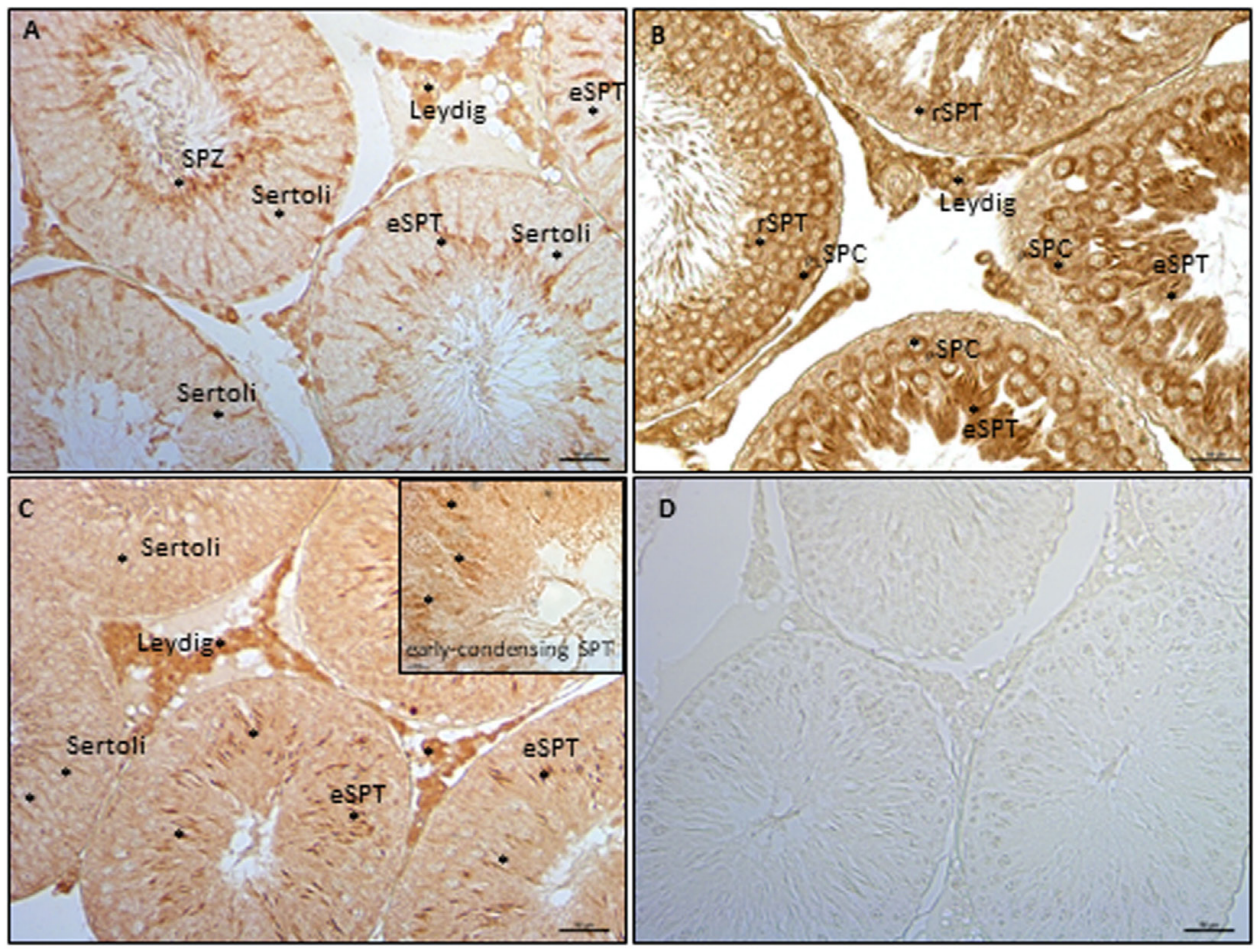
Immunocytochemistry analyses of endocannabinoid system components in testis from rat at 90 *days post partum*. Immunolocalization of NAPE-PLD **(A)**, fatty acid amide hydrolase **(B)**, and monoacylglycerol-lipase **(C)** proteins in interstitial and tubular compartments. Asterisks indicate immunopositive cells. Specificity of reaction was checked by omitting the primary antibody **(D)**. Scale bar: 50 µm; inset scale bar: 10 µm.

The expression of ECS components in germ cells has been analyzed in rat testis by ICC, using testis from 90 *dpp* rat. In particular, have been analyzed NAPE-PLD, FAAH, and MAGL proteins. ICC analysis show NAPE-PLD, FAAH, and MAGL proteins in Leydig cells (Figure 5). In tubular compartment, NAPE-PLD was feebly present in Sertoli cells and highly expressed in SPT and SPZ (Figure 5A). FAAH was present in germ cells, from preleptotene and pachytene SPC (_PL_SPC and _P_SPC) to round (r) and elongated (e) SPT (Figure 5B) while MAGL (Figure 5C) was feebly present in Sertoli cells associated to round SPT (stage IX/X), and in early condensing SPT (inset). Higher immunolocalization was observed in elongating SPT (eSPT).

### Functional Interaction Among ECS Components

**Figure 6:**
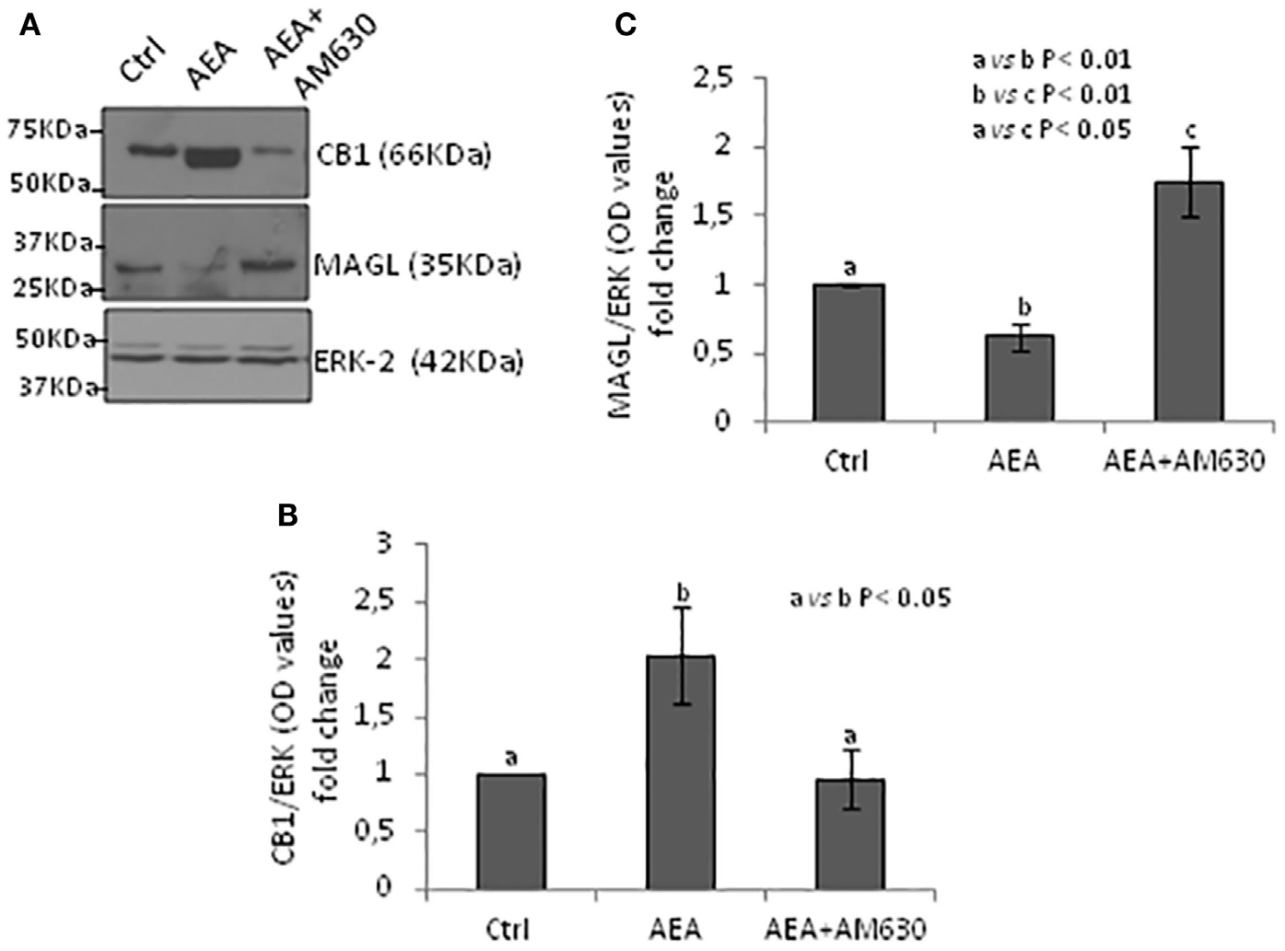
Responsiveness of monoacylglycerol-lipase (MAGL) to CB1/CB2 receptors. Western blot analysis of CB1 and MAGL in CB1^+/−^ testes exposed to vehicle or AEA ± AM630 **(A)**. CB1 amount was quantified by densitometry analysis, normalized against ERK1/2 signals, and expressed in OD values as fold change **(B)**. MAGL amount was quantified by densitometry analysis, normalized against ERK1/2 signals, and expressed in OD values as fold change **(C)**. All data are reported as mean ± SEM.

Testes from mice null of CB1 gene in heterozygous conditions (CB1^+/−^) were used as model tissue of low levels of CB1 to verify responsiveness of expression of CB1 and MAGL proteins to AEA/CB2 activity. Testes CB1^+/−^ were incubated with vehicle or AEA, in presence/absence of AM630, a selective inverse agonist for CB2, and processed to quantify CB1 and MAGL proteins by Western blot analysis. Results are reported in Figure 6. Densitometry analysis of signals show that AEA significantly (*p* < 0.05) increased CB1 protein levels (Figure 6B) in comparison to control group, while a negative effect was observed (*p* < 0.01) on MAGL protein levels (Figure 6C). Both AEA-induced effects were efficiently counteracted by AM630 with different levels of statistical significance (*p* < 0.05 and *p* < 0.01).

## Discussion

The presence of ECS components has been investigated in rat testis during the first wave of spermatogenesis, from 7 to 60 *dpp*, to analyze the fluctuations of enzymes and receptors with respect to mitotic, meiotic, and post-meiotic germ cells. The expression profiles of the main enzymes metabolizing AEA and 2-AG (i.e., *Faah, Nape-pld, Magl, Dagl*), as well as the expression of CB receptors (*Cb1, Cb2*) and specific markers of germ cell presence or activity have been analyzed by RT-PCR and appropriately correlated.

Results showed detailed fluctuations of gene expression. *Faah* and Nape-pld were poorly expressed at 7 *dpp*. Thereafter, *Faah* progressively and significantly increased up to 60 *dpp* while *Nape-pld* increased later, starting from 27 *dpp*. These data suggested that *Faah* expression was related to germ cell progression from SPG-to-SPT, while *Nape-pld* expression was more specifically related to SPT appearance. The high levels of *Faah* in SPC and SPT (*mRNA* and/or protein), when compared with SPG, have already been demonstrated in both frog and mouse (46, 50) and here confirmed by ICC analysis. Interestingly, *Faah* was consistently higher as compared with *Nape-pld*, except at 7 and 41 *dpp*, when similar *Nape-pld/Faah mRNA* levels were observed. This suggested that, during spermatogenesis progression, *Faah* was constantly expressed to control intra-testicular AEA levels, and that peaks of AEA specifically occurred in the testis during early and later phases of spermatogenesis. However, the expression profile of *Dagl* and *Magl* clearly showed that early phases of spermatogenesis required high intra-testicular 2-AG levels. *Vice versa*, lower amounts of 2-AG were necessary later. Accordingly, data from isolated mouse germ cells demonstrate that SPG produce more 2-AG due to the higher *Dagl* and lower *Magl* expression (both *mRNA* and protein) when compared with meiotic and post-meiotic cells (50). In agreement, ICC analysis show that testis from rat at 90 *dpp* expressed MAGL in Leydig cells and appeared in tubular compartment later during spermatogenesis in Sertoli cells and SPT.

The expression profile of CB1 in rat testis, already investigated by our group during the first wave of spermatogenesis (49), was here analyzed in fetal and post-natal testis, with respect to gonocyte activity during their mitotic arrest (i.e., in G1 phase) and proliferative resumption. More specifically, we immunolocalized CB1 protein in fetal testis during proliferation (14.5 *dpc*) and mitotic arrest in G1 phase (19.5 *dpc*) of gonocytes, then we studied testicular fluctuations of *Cb1 mRNA* in *post-natal* testis, during the cell cycle arrest (1 *dpp*), mitotic resumption (7 *dpp*), and meiotic entry (14 *dpp*). The expression of *Cxcl12* (66) and *Mlh3* (67) was used to specifically define the activity of the SPG stem cell pool maintenance and the appearance of SPC, respectively. In addition, we studied the expression profile of *Cb2*, from 7 to 60 *dpp*, using *Hsp70t* (68) to specifically define SPT appearance from 21 *dpp* forward.

Data reported here show that CB1 was present in the testis at 19.5 *dpc*. The protein was exclusively localized in gonocytes. The lack of signals at 14.5 *dpc*, suggested that CB1 protein was functionally related to mitotic G1 arrest of germ cells (5) confirming the cytostatic activity of CB1 observed in Leydig cells (39). In agreement with data from mouse and rat (45, 49), the gene expression analysis carried out on post-natal testis revealed that *Cb1 mRNA* levels were low at 1 and 14 *dpp* and high at 7 *dpp*. Indeed, *Cb1* profile well correlated with *Cxcl12* profile and both matched specifically with post-natal fluctuations of AEA metabolizing enzymes suggesting that CB1 and a weak intra-testicular AEA rise were related to germ cell mitotic resumption. More specifically, the significant correlation of *Cb1* with *Cxcl12* suggested a potential involvement of CB1 in SPG stem cell pool maintenance activity (i.e., self-renewal activity and/or the relative commitment). However, *Cb1 mRNA* decreased at 14 *dpp* when type-A SPG were present in the testis and proliferating to become SPC. Interestingly, the impressive *Cb1* decrease was associated with the appearance of *Mlh3* which expression increased in correlation with *Cb2* and in presence of high *Dagl* and low *Magl mRNA* levels. This suggests that SPG proliferation and meiotic entry required the presence of CB2 and high levels of 2-AG. In agreement, Grimaldi and coworkers demonstrated that CB2 is highly expressed in mouse SPG, and its activation, likely *via* 2-AG, promotes meiotic entry (50). However, during later stages of spermatogenesis, at 27 *dpp*, the expression of ECS components changed drastically, in concomitance with the appearance of SPT. Indeed, the expression profile of *Hsp70t* demonstrated that SPT appeared between 21 and 31 *dpp* enriching progressively the germinal compartment. Simultaneously, a significant and gradual increase of *Nape-pld* and *Magl* was observed suggesting that, when in the testis *post*-meiotic germ cells are present (49), these cells might be or support the AEA source and play a key role in 2-AG degradation.

Data obtained in mouse show that SPT produce appreciable amount of AEA and that 2-AG is more efficiently synthesized in SPG when compared with SPC and SPT (50). Consistently, SPT result also to be the main producers of Magl, among germ cells (50). To verify these data in our animal model, we carried out correlation analyses within the appropriate time frame, 21–60 *dpp*. Results showed that *Nape-pld, Faah*, and *Magl* fluctuations correlated positively with *Hsp70t* expression. In agreement, ICC analysis confirm higher expression of NAPE-PLD and FAAH proteins from round to elongated SPT, while MAGL feebly appeared in early condensing SPT and Sertoli cells and higher expressed in elongating SPT. A negative relation was observed when *Dagl/Magl mRNA* ratio, here used as indirect index of intra-testicular 2-AG levels, was correlated to expression of *Hsp70t*. The expression profiles of *Nape-pld, Magl*, and *Cb2* were positively correlated each other. We concluded that the appearance of SPT was directly or indirectly responsible of AEA synthesis and 2-AG hydrolysis.Studies carried out on murine isolated germ cells and immature mouse demonstrate that CB2 signaling, likely responsive to 2-AG, controls meiotic entry (SPG/preleptotene-SPC differentiation) of SPG (50, 59). Interestingly, spermatogenic progression of more immature germ cells is responsive to feedback signals from more mature germ cells (13). Indeed, an intriguing testicular network of cell-to-cell communication controls progression of more mature and immature germ cells, properly clustered in specific stages. Intriguingly, in immature rat testis, CB1 appears in Sertoli cells in combination with the appearance of elongated SPT (22, 49). In mature rat testis, a weak and stage-specific expression of CB1 has been observed in Sertoli cells at stages VIII–IX/X (49) corresponding to Sertoli/SPC/elongated SPT-clustered cells. In frog and rat, the presence of SPT is positively associated with the increase of CB1 (46, 49). In frog testis, CB1 expression is responsive to AEA (69). Therefore, we hypothesized that SPT control synthesis of AEA and that such a ligand, through activation of both CB receptors, affects 2-AG levels promoting meiotic entry of SPC. With this model in mind, we checked at molecular level, if AEA, *via* CB2, downregulated MAGL by inducing CB1 increase. Testis from mice null of CB1 in heterozygous condition (CB1^+/−^) was used as model tissue of down-expression of CB1 (then potentially responsive to increase after stimulation) and stimulated with AEA ± AM630. The aim was to verify the responsiveness of CB1 and MAGL protein expression to AEA/CB2 signaling. Results demonstrate that AEA-CB2 activity affected negatively MAGL levels *via* upregulation of CB1. This functional interaction of ECS components supports data above reported (i.e., low levels of *Magl* are present in the testis during mitotic/meiotic cell progression; high levels of *Nape-pld* are present in the testis when post-meiotic cells appear) and suggest their functional interaction (i.e., functional clustering) in testis during germ cell progression demonstrating a CB1/CB2-mediated relationship between AEA and 2-AG. Intriguingly, this might be the molecular network through which the appearance of elongated SPT control meiotic entry of SPC (13). Of course, further analyses should be specifically addressed to verify this model of cell-to-cell communication.

Altogether, our data perfectly matches those obtained in isolated mouse germ cells (50) thus excluding species-specific differences (mouse vs rat) and reinforcing the rational about our methodological choice (see Materials and Methods). Further analyses are necessary to specifically characterize the cells expressing CB receptors and enzymes metabolizing AEA and 2-AG to better define cellular network involved in local control of spermatogenesis. However, the above described observations are relevant since these show that *mRNA* level variations of ECS components are extremely robust, highly conserved, and functionally clustered during spermatogenesis. Accordingly, our data should be considered as identifying good molecular markers to follow a correct spermatogenesis progression. In this context, present data may open the possibility to develop algorithms for describing and monitoring testicular function. In the literature, spermatogenesis computational analysis, based on whole transcriptome, has already been described (70, 71). However, algorithms based on the expression levels of functionally clustered genes (as ECS components) might be a better and simpler way to monitor this complex event.

In conclusion, data here reported show that ECS components are functionally clustered and are differentially related to germ cell progression. In particular, CB2 and 2-AG appear to be related to mitotic/meiotic stages, while CB1 and AEA appear to be related to SPG stem cells activity and SPT appearance, respectively. We show that CB2 activity, stimulated *in vitro* by AEA, affects MAGL levels *via* upregulation of CB1, providing the first functional data supporting a CB1/CB2-mediated relationship between AEA and 2-AG. Furthermore, we show that fetal testis expresses CB1 providing, for the first time, evidence that the protein is present in gonocytes blocked in mitosis. Finally, present data may shed light in the study of complex systems computational modeling.

## Ethics Statement

Experiment were approved by the Italian Ministry of Education (MIUR) and the Italian Ministry of Health. All methods and all animal procedures were performed in accordance with the relevant guidelines and regulations by National Research Council’s (NRC) for Care and Use of Laboratory Animals (NIH Guide).

## Author Contributions

TC, MM, GR, FM, and GC: conception and design of the work. TC and GC: manuscript drafting; AS: figures preparation. KM and GC: critical revision. SF and RP: final version approval.

## Conflict of Interest Statement

The authors declare that the research was conducted in the absence of any commercial or financial relationships that could be construed as a potential conflict of interest.

## References

[1] Cobellis G Noviello C Nino F Romano M Mariscoli F Martino A Spermatogenesis and cryptorchidism. Front Endocrinol (2014) 5:63. 10.3389/fendo.2014.00063 24829558 PMC4013472

[2] Ricci G Catizone A Galdieri M. Pleiotropic activity of hepatocyte growth factor during embryonic mouse testis development. Mech Dev (2002) 118:19–28. 10.1016/S0925-4773(02)00247-2 12351166

[3] Ricci G Catizone A Galdieri M Embryonic mouse testis development: role of platelet derived growth factor (PDGF-BB). J Cell Physiol (2004) 200:458–67. 10.1002/jcp.20035 15254974

[4] Spiller CM Feng CW Jackson A Gillis AJ Rolland AD Looijenga LH Endogenous nodal signaling regulates germ cell potency during mammalian testis development. Development (2012) 139:4123–32. 10.1242/dev.083006 23034635

[5] Moreno SG Dutrillaux B Coffigny H. Study of the gonocyte cell cycle in irradiated TP53 knockout mouse foetuses and newborns. Int J Radiat Biol (2002) 78:703–9. 10.1080/09553000210134818 12194754

[6] Hess RA Spermatogenesis: an overview. In: Knobil E Neill JD, editors. Encyclopedia of Reproduction. San Diego; London; Boston; New York; Sydney; Tokyo; Toronto: Academic Press (1999). p. 539–45.

[7] Cacciola G Chioccarelli T Fasano S Pierantoni R Cobellis G Estrogens and spermiogenesis: new insights from type 1 cannabinoid receptor knockout mice. Int J Endocrinol (2013) 2013:501350 10.1155/2013/501350 24324492 PMC3845505

[8] Sharpe RM. Paracrine control of the testis. Clin Endocrinol Metab (1986) 15:185–207. 10.1016/S0300-595X(86)80049-4 3514003

[9] Cobellis G Pierantoni R Minucci S Pernas-Alonso R Meccariello R Fasano S. c-fos activity in Rana esculenta testis: seasonal and estradiol-induced changes. Endocrinology (1999) 140:3238–44. 10.1210/endo.140.7.6790 10385420

[10] O’Donnell L Robertson KM Jones ME Simpson ER. Estrogen and spermatogenesis. Endocr Rev (2001) 22:289–318. 10.1210/er.22.3.289 11399746

[11] Cobellis G Meccariello R Fienga G Pierantoni R Fasano S. Cytoplasmic and nuclear Fos protein forms regulate resumption of spermatogenesis in the frog, Rana esculenta. Endocrinology (2002) 143:163–70. 10.1210/endo.143.1.8567 11751605

[12] Pierantoni R Cobellis G Meccariello R Palmiero C Fienga G Minucci S The amphibian testis as model to study germ cell progression during spermatogenesis. Comp Biochem Physiol B Biochem Mol Biol (2002) 132:131–9. 10.1016/S1096-4959(01)00543-7 11997216

[13] Pierantoni R Cobellis G Meccariello R Fasano S Evolutionary aspects of cellular communication in the vertebrate hypothalamo-hypophysio-gonadal axis. Int Rev Cytol (2002) 218:69–141. 10.1016/S0074-7696(02)18012-0 12199520

[14] Cobellis G Meccariello R Minucci S Palmiero C Pierantoni R Fasano S. Cytoplasmic versus nuclear localization of Fos-related proteins in the frog, Rana esculenta, testis: in vivo and direct in vitro effect of a gonadotropin-releasing hormone agonist. Biol Reprod (2003) 68:954–60. 10.1095/biolreprod.102.008938 12604648

[15] Cobellis G Lombardi M Scarpa D Izzo G Fienga G Meccariello R Fra1 activity in the frog, Rana esculenta, testis: a new potential role in sperm transport. Biol Reprod (2005) 72:1101–8. 10.1095/biolreprod.104.036541 15625234

[16] Catizone A Ricci G Del Bravo J Galdieri M. Hepatocyte growth factor modulates in vitro survival and proliferation of germ cells during postnatal testis development. J Endocrinol (2006) 191:559–70. 10.1677/joe.1.06528 16614388

[17] Carreau S Bouraima-Lelong H Delalande C Estrogens: new players in spermatogenesis. Repr Biol (2011) 11:174–93. 10.1016/S1642-431X(12)60065-5 22139333

[18] Chianese R Chioccarelli T Cacciola G Ciaramella V Fasano S Pierantoni R The contribution of lower vertebrate animal models in human reproduction research. Gen Comp Endocrinol (2011) 171:17–27. 10.1016/j.ygcen.2010.12.011 21192939

[19] Meccariello R Fasano S Pierantoni R Cobellis G Modulators of hypothalamic-pituitary-gonadal axis for the control of spermatogenesis and sperm quality in vertebrates. Front Endocrinol (2014) 5:135 10.3389/fendo.2014.00135 PMC4135230 25183961

[20] Meccariello R Chianese R Chioccarelli T Ciaramella V Fasano S Pierantoni R Intra-testicular signals regulate germ cell progression and production of qualitatively mature spermatozoa in vertebrates. Front Endocrinol (2014) 5:69 10.3389/fendo.2014.00069 PMC4021137 24847312

[21] Wenger T Ledent C Csernus V Gerendai I. The central cannabinoid receptor inactivation suppresses endocrine reproductive functions. Biochem Biophys Res Commun (2001) 284:363–8. 10.1006/bbrc.2001.4977 11394887

[22] Pierantoni R Cobellis G Meccariello R Cacciola G Chianese R Chioccarelli T CB1 activity in male reproduction: mammalian and nonmammalian animal models. Vitam Horm (2009) 81:367–87. 10.1016/S0083-6729(09)81014-5 19647119

[23] Pierantoni R Cobellis G Meccariello R Cacciola G Chianese R Chioccarelli T Testicular gonadotropin-releasing hormone activity, progression of spermatogenesis, and sperm transport in vertebrates. Ann N Y Acad Sci (2009) 1163:279–91. 10.1111/j.1749-6632.2008.03617.x 19456349

[24] Battista N Meccariello R Cobellis G Fasano S Di Tommaso M Pirazzi V The role of endocannabinoids in gonadal function and fertility along the evolutionary axis. Mol Cell Endocrinol (2012) 355:1–14. 10.1016/j.mce.2012.01.014 22305972

[25] Grimaldi P Di Giacomo D Geremia R. The endocannabinoid system and spermatogenesis. Front Endocrinol (2013) 4:192. 10.3389/fendo.2013.00192 24379805 PMC3864102

[26] Wang H Dey SK Maccarrone M. Jekyll and Hyde: two faces of cannabinoid signaling in male and female fertility. Endocr Rev (2006) 27:427–48. 10.1210/er.2006-0006 16682502

[27] Acone G Trabucco E Colacurci N Cobellis L Mackie K Meccariello R Low type I cannabinoid receptor levels characterize placental villous in labouring delivery. Placenta (2009) 30:203–5. 10.1016/j.placenta.2008.11.018 19097644

[28] Trabucco E Acone G Marenna A Pierantoni R Cacciola G Chioccarelli T Endocannabinoid system in first trimester placenta: low FAAH and high CB1 expression characterize spontaneous miscarriage. Placenta (2009) 30:516–22. 10.1016/j.placenta.2009.03.015 19419760

[29] Matsuda LA Lolait SJ Brownstein MJ Young AC Bonner TI. Structure of a cannabinoid receptor and functional expression of the cloned cDNA. Nature (1990) 346:561–4. 10.1038/346561a0 2165569

[30] Munro S Thomas KL Abu-Shaar M. Molecular characterization of a peripheral receptor for cannabinoids. Nature (1993) 365:61–5. 10.1038/365061a0 7689702

[31] Devane WA Hanus L Breuer A Pertwee RG Stevenson LA Griffin G Isolation and structure of a brain constituent that binds to the cannabinoid receptor. Science (1992) 258:1946–9. 10.1126/science.1470919 1470919

[32] Sugiura T Kondo S Sukagawa A Nakane S Shinoda A Itoh K 2-Arachidonoylglycerol: a possible endogenous cannabinoid receptor ligand in brain. Biochem Biophys Res Commun (1995) 215:89–97. 10.1006/bbrc.1995.2437 7575630

[33] Okamoto Y Morishita J Tsuboi K Tonai T Ueda N. Molecular characterization of a phospholipase D generating anandamide and its congeners. J Biol Chem (2004) 279:5298–305. 10.1074/jbc.M306642200 14634025

[34] Bisogno T Howell F Williams G Minassi A Cascio MG Ligresti A Cloning of the first sn1-DAG lipases points to the spatial and temporal regulation of endocannabinoid signaling in the brain. J Cell Biol (2003) 163:463–8. 10.1083/jcb.200305129 14610053 PMC2173631

[35] Dinh TP Carpenter D Leslie FM Freund TF Katona I Sensi SL Brain monoglyceride lipase participating in endocannabinoid inactivation. Proc Natl Acad Sci U S A (2002) 99:13961. 10.1073/pnas.152334899 12136125 PMC125056

[36] McKinney MK Cravatt BF. Structure and function of fatty acid amide hydrolase. Annu Rev Biochem (2005) 74:411–32. 10.1146/annurev.biochem.74.082803.133450 15952893

[37] Iannotti FA Silvestri C Mazzarella E Martella A Calvigioni D Piscitelli F The endocannabinoid 2-AG controls skeletal muscle cell differentiation via CB1 receptor-dependent inhibition of Kv7 channels. Proc Natl Acad Sci U S A (2014) 111:E2472–81. 10.1073/pnas.1406728111 24927567 PMC4066524

[38] Suglia A Chianese R Migliaccio M Ambrosino C Fasano S Pieranton R Bisphenol A induces hypothalamic down-regulation of the cannabinoid receptor 1 and anorexigenic effects in male mice. Pharmacol Res (2016) 113:376–83. 10.1016/j.phrs.2016.09.005 27641926

[39] Cacciola G Chioccarelli T Ricci G Meccariello R Fasano S Pierantoni R The endocannabinoid system in vertebrate male reproduction: a comparative overview. Mol Cell Endocrinol (2008) 286:S24–30. 10.1016/j.mce.2008.01.004 18342433

[40] Bovolin P Cottone E Pomatto V Fasano S Pierantoni R Cobellis G Endocannabinoids are involved in male vertebrate reproduction: regulatory mechanisms at central and gonadal level. Front Endocrinol (2014) 5:54. 10.3389/fendo.2014.00054 24782832 PMC3995072

[41] Cacciola G Chianese R Chioccarelli T Ciaramella V Fasano S Pierantoni R Cannabinoids and reproduction: a lasting and intriguing history. Pharmaceuticals (2010) 3:3275–323. 10.3390/ph3103275

[42] Pertwee RG Joe-Adigwe G Hawksworth GM. Further evidence for the presence of cannabinoid CB1 receptors in mouse vas deferens. Eur J Pharmacol (1996) 296:169–72. 10.1016/0014-2999(95)00790-3 8838453

[43] Maccarrone M Cecconi S Rossi G Battista N Pauselli R Finazzi-Agro A. Anandamide activity and degradation are regulated by early postnatal aging and follicle-stimulating hormone in mouse Sertoli cells. Endocrinology (2003) 144:20–8. 10.1210/en.2002-220544 12488326

[44] Maccarrone M Barboni B Paradisi A Bernabò N Gasperi V Pistilli MG Characterization of the endocannabinoid system in boar spermatozoa and implications for sperm capacitation and acrosome reaction. J Cell Sci (2005) 118:4393–404. 10.1242/jcs.02536 16144868

[45] Gye MC Kang HH Kang HJ. Expression of cannabinoid receptor 1 in mouse testes. Arch Androl (2005) 51:247–55. 10.1080/014850190898845 16025865

[46] Cobellis G Cacciola G Scarpa D Meccariello R Chianese R Franzoni MF Endocannabinoid system in frog and rodent testis: type-1 cannabinoid receptor and fatty acid amide hydrolase activity in male germ cells. Biol Reprod (2006) 75:82–9. 10.1095/biolreprod.106.051730 16611862

[47] Ricci G Cacciola G Altucci L Meccariello R Pierantoni R Fasano S Endocannabinoid control of sperm motility: the role of epididymus. Gen Comp Endocrinol (2007) 153:320–2. 10.1016/j.ygcen.2007.02.003 17395184

[48] Rossi G Gasperi V Paro R Barsacchi D Cecconi S Maccarrone M. Follicle-stimulating hormone activates fatty acid amide hydrolase by protein kinase A and aromatase-dependent pathways in mouse primary Sertoli cells. Endocrinology (2007) 148:431–9. 10.1210/en.2006-0969 17110429

[49] Cacciola G Chioccarelli T Mackie K Meccariello R Ledent C Fasano S Expression of type-1 cannabinoid receptor during rat postnatal testicular development: possible involvement in adult Leydig cell differentiation. Biol Reprod (2008) 79:758–65. 10.1095/biolreprod.108.070128 18614700

[50] Grimaldi P Orlando P Di Siena S Lolicato F Petrosino S Bisogno T The endocannabinoid system and pivotal role of the CB2 receptor in mouse spermatogenesis. Proc Natl Acad Sci U S A (2009) 106:11131–6. 10.1073/pnas.0812789106 19541620 PMC2708706

[51] Cobellis G Ricci G Cacciola G Orlando P Petrosino S Cascio MG A gradient of 2-arachidonoylglycerol regulates mouse epididymal sperm cell start-up. Biol Reprod (2010) 82:451–8. 10.1095/biolreprod.109.079210 19812302

[52] Rossato M Ion Popa F Ferigo M Clar IG Foresta C. Human sperm express cannabinoid receptor Cb1, the activation of which inhibits motility, acrosome reaction, and mitochondrial function. J Clin Endocrinol Metab (2005) 90:984–91. 10.1210/jc.2004-1287 15562018

[53] Chianese R Ciaramella V Scarpa D Fasano S Pierantoni R Meccariello R. Anandamide regulates the expression of GnRH1, GnRH2, and GnRH-Rs in frog testis. Am J Physiol (2012) 303:E475–87. 10.1152/ajpendo.00086.2012 22669247

[54] Cacciola G Chioccarelli T Altucci L Ledent C Mason JI Fasano S Low 17beta-estradiol levels in Cnr1 knock-out mice affect spermatid chromatin remodeling by interfering with chromatin reorganization. Biol Reprod (2013) 88:1–12. 10.1095/biolreprod.112.105726 23677985

[55] Cacciola G Chioccarelli T Altucci L Viggiano A Fasano S Pierantoni R Nuclear size as estrogen-responsive chromatin quality parameter of mouse spermatozoa. Gen Comp Endocrinol (2013) 193:201–9. 10.1016/j.ygcen.2013.07.018 23973938

[56] Cobellis G Meccariello R Chianese R Chioccarelli T Fasano S Pierantoni R. Effects of neuroendocrine CB1 activity on adult Leydig cells. Front Endocrinol (2016) 7:47. 10.3389/fendo.2016.00047 27375550 PMC4891325

[57] Chioccarelli T Cacciola G Altucci L Lewis SE Simon L Ricci G Cannabinoid receptor 1 influences chromatin remodeling in mouse spermatids by affecting content of transition protein 2 mRNA and histone displacement. Endocrinology (2010) 151:5017–29. 10.1210/en.2010-0133 20810562

[58] Francavilla F Battista N Barbonetti A Vassallo MR Rapino C Antonangelo C Characterization of the endocannabinoid system in human spermatozoa and involvement of transient receptor potential vanilloid 1 receptor in their fertilizing ability. Endocrinology (2009) 150:4692–700. 10.1210/en.2009-0057 19608651

[59] Di Giacomo D De Domenico E Sette C Geremia R Grimaldi P. Type 2 cannabinoid receptor contributes to the physiological regulation of spermatogenesis. FASEB J (2016) 30:1453–63. 10.1096/fj.15-279034 26671998

[60] Ledent C Valverde O Cossu G Petitet F Aubert JF Beslot F Unresponsiveness to cannabinoids and reduced addictive effects of opiates in CB1 receptor knockout mice. Science (1999) 283:401–4. 10.1126/science.283.5400.401 9888857

[61] Santangeli S Maradonna F Gioacchini G Cobellis G Piccinetti CC Dalla Valle L BPA-induced deregulation of epigenetic patterns: effects on female zebrafish reproduction. Sci Rep (2016) 6:21982. 10.1038/srep21982 26911650 PMC4766405

[62] Crispi S Calogero RA Santini M Mellone P Vincenzi B Citro G Global gene expression profiling of human pleural mesotheliomas: identification of matrix metalloproteinase 14 (MMP-14) as potential tumour target. PLoS One (2009) 4:e7016. 10.1371/journal.pone.0007016 19753302 PMC2737627

[63] Hsieh C Brown S Derleth C Mackie K. Internalization and recycling of the CB1 cannabinoid receptor. J Neurochem (1999) 73:493–501. 10.1046/j.1471-4159.1999.0730493.x 10428044

[64] Ross RA Brockie HC Stevenson LA Murphy VL Templeton F Makriyannis A Agonist-inverse agonist characterization at CB1 and CB2 cannabinoid receptors of L759633, L759656, and AM630. Br J Pharmacol (1999) 126:665–72. 10.1038/sj.bjp.0702351 10188977 PMC1565857

[65] Bolognini D Cascio MG Parolaro D Pertwee RG. AM630 behaves as a protean ligand at the human cannabinoid CB2 receptor. Br J Pharmacol (2012) 165:2561–74. 10.1111/j.1476-5381.2011.01503.x 21615724 PMC3423246

[66] Yang QE Kim D Kaucher A Oatley MJ Oatley JM. CXCL12-CXCR4 signaling is required for the maintenance of mouse spermatogonial stem cells. J Cell Sci (2013) 126:1009–20. 10.1242/jcs.119826 23239029 PMC4074255

[67] Lipkin SM Moens PB Wang V Lenzi M Shanmugarajah D Gilgeous A Meiotic arrest and aneuploidy in MLH3-deficient mice. Nat Genet (2002) 31:385–90. 10.1038/ng931 12091911

[68] Ito Y Ando A Ando H Ando J Saijoh Y Inoko H Genomic structure of the spermatid-specific hsp70 homolog gene located in the class III region of the major histocompatibility complex of mouse and man. J Biochem (1998) 124:347–53. 10.1093/oxfordjournals.jbchem.a022118 9685725

[69] Ciaramella V Meccariello R Chioccarelli T Sirleto M Fasano S Pierantoni R Anandamide acts via kisspeptin in the regulation of testicular activity of the frog, Pelophylax esculentus. Mol Cell Endocrinol (2016) 420:75–84. 10.1016/j.mce.2015.11.011 26586207

[70] Cappallo-Obermann H Feig C Schulze W Spiess AN. Fold-change correction values for testicular somatic transcripts in gene expression studies of human spermatogenesis. Hum Reprod (2013) 28:590–8. 10.1093/humrep/des433 23303554

[71] Margolin G Khil PP Kim J Bellani MA Camerini-Otero RD. Integrated transcriptome analysis of mouse spermatogenesis. BMC Genomics (2014) 15:39. 10.1186/1471-2164-15-39 24438502 PMC3906902

